# Short-Term Periodic Fasting Reduces Ischemia-Induced Necrosis in Musculocutaneous Flap Tissue

**DOI:** 10.3390/biomedicines12030690

**Published:** 2024-03-20

**Authors:** Andrea Weinzierl, Maximilian Coerper, Yves Harder, Michael D. Menger, Matthias W. Laschke

**Affiliations:** 1 Institute for Clinical and Experimental Surgery, Saarland University, 66421 Homburg, Germany; 2 Department of Plastic Surgery and Hand Surgery, University Hospital Zurich, 8091 Zurich, Switzerland; 3 Department of Plastic, Reconstructive and Aesthetic Surgery, Ospedale Regionale di Lugano, Ente Ospedaliero Cantonale (EOC), 6900 Lugano, Switzerland; 4 Faculty of Biomedical Sciences, Università della Svizzera Italiana, 6900 Lugano, Switzerland

**Keywords:** dietary restriction, periodic fasting, necrosis, angiogenesis, nutrition, microcirculation

## Abstract

Periodic fasting (PF) as a form of dietary restriction has been shown to induce tissue-protective effects against ischemic injury in several different tissues. Accordingly, in this study we analyzed whether a short-term 24 h fast is suitable to prevent necrosis of musculocutaneous flap tissue undergoing acute persistent ischemia. C57BL/6N mice were randomly divided into a PF group (*n* = 8) and a control group that was given unrestricted access to standard chow (*n* = 8). The PF animals underwent a 24 h fast immediately before flap elevation and had unrestricted access to food for the rest of the 10 day observation period. Musculocutaneous flaps with a random pattern design were dissected on the animals’ backs and mounted into dorsal skinfold chambers. On days 1, 3, 5, 7 and 10 after surgery, nutritive tissue perfusion, angiogenesis and flap necrosis were evaluated using intravital fluorescence microscopy. Thereafter, the flap tissue was excised and fixed for histological and immunohistochemical analyses. The flaps of PF-treated animals exhibited a higher functional capillary density and more newly formed microvessels, resulting in a significantly increased flap survival rate. Moreover, they contained a lower number of myeloperoxidase (MPO)-positive neutrophilic granulocytes and cleaved caspase-3-positive apoptotic cells in the transition zone between vital and necrotic flap tissue. These findings indicate that short-term PF improves tissue survival in ischemically challenged musculocutaneous flaps by maintaining nutritive blood perfusion and dampening ischemia-induced inflammation.

## 1. Introduction

When dealing with large surface tissue defects, plastic surgeons often rely on tissue flaps to reconstruct the surface and shape of the human body. Whenever possible, a designated vascular axis that constitutes the pedicle of the flap is included in an axial pattern flap to supply robust perfusion. Alternatively, flaps may be raised using a random pattern design [1]. In this case, nutritive blood perfusion is provided by musculocutaneous arterioles passing through the flap’s base and/or the dermal plexus. The limited arterial inflow can leave the flap’s periphery at risk for ischemic complications, including wound breakdown or necrosis, as may be the case in extended pedicled flaps or microvascular flaps that include tissue areas beyond their angiosome. In fact, hypoxia and nutrient deprivation occur due to the insufficient tissue perfusion, resulting in cellular dysfunction and ischemic cell death. To avoid ischemia-induced complications, flaps are specifically designed considering different factors, such as thickness and tissue composition, length-to-width-ratio as well as absolute length and capillary closing pressure [2,3]. However, ischemia-induced complications still occur with highly relevant rates of up to 37.5% [4].

Various strategies to reduce this type of complication have been investigated in the past [5,6], as wound break down and tissue necrosis is associated with a higher patient morbidity and a substantial socio-economic burden due to added treatment costs. One of the earliest concepts was the so called “surgical delay”, either by means of stepwise flap elevation [7], or by ligating selective feeding vessels to the flap territory (“selective delay”) [8]. More recently, dietary restriction (DR) has been proposed as a promising approach to increase tissue survival [9]. DR is an attractive option for perioperative treatment, as it has almost no unwanted side effects and few contraindications, such as malnourished patients and patients prone to hypoglycemia [10]. Typical forms of DR include time-restricted eating (TRE), fasting-mimicking diets (FMD), intermittent fasting (IF; shorter and more frequently repeated fasts) and periodic fasting (PF; longer, less frequently or unrepeated fasts) [11]. As there is no systematic nomenclature, fasting protocols may overlap or be referred to under different names.

Initial DR research concerned healthy aging and life span extension [12]. These evolutionarily conserved effects may be seen as the remnant of mechanisms to increase tissue resistance in an environment with limited energy resources and are already present in prokaryotes [13]. While life span extension can only be achieved by applying long-term DR [14], DR is also protective against acute stress events after short-term dietary interventions [15]. Due to the organism’s ability to respond rapidly to changes in nutritional intake, short-term DR prior to ischemia prevents negative ischemic effects; for instance, by suppressing inflammation [16]. Accordingly, DR has been shown to counteract hypoxic stress in ischemic myocardial, renal, hepatic and cerebral tissue [17,18,19,20]. For instance, overall survival and kidney function in mice were significantly improved after the application of only 2 weeks of 30% caloric restriction (CR) before the induction of renal ischemia [21]. Similarly, a 16 h fast prior to myocardial ischemia in rats decreased cellular damage and improved the recovery of myocardial function [22].

These beneficial effects of DR have also been observed in surgical flaps. In a recent study, we reported that a 10 day period of IF markedly increases the survival of musculocutaneous random pattern flaps in mouse dorsal skinfold chambers [23]. However, this period may already be too long to successfully implement the DR protocol into clinical routines. The aim of the present study was therefore to evaluate, whether a short-term 24 h fast immediately before surgical flap elevation is also able to effectively reduce ischemia-induced tissue necrosis.

## 2. Materials and Methods

### 2.1. Animals

The animal experiments conducted for the present study were reviewed and approved by the local governmental animal protection committee (permit number: 10/2020 with the Office for Consumer Protection, Saarbrücken, Germany). The study was carried out in conformity with the European legislation on the protection of animals (Directive 2010/63/EU) and the NIH Guidelines on the Care and Use of Laboratory Animals (NIH publication #85-23 Rev. 1985).

Sixteen C57BL/6N mice (Institute for Clinical and Experimental Surgery, Saarland University, Homburg, Germany) were used for the experiments at an age of 12–24 weeks with a body weight of 26–30 g. The mice were kept one per cage at a room temperature of 22–24 °C, a relative humidity of 50–60% and a 12 h day–night cycle for the duration of the in vivo observation period. The animals had unrestricted access to standard pellet chow (Altromin, Lage, Germany) and tap water except for the animals treated with a fasting protocol.

### 2.2. PF Regimen

**Figure 1:**
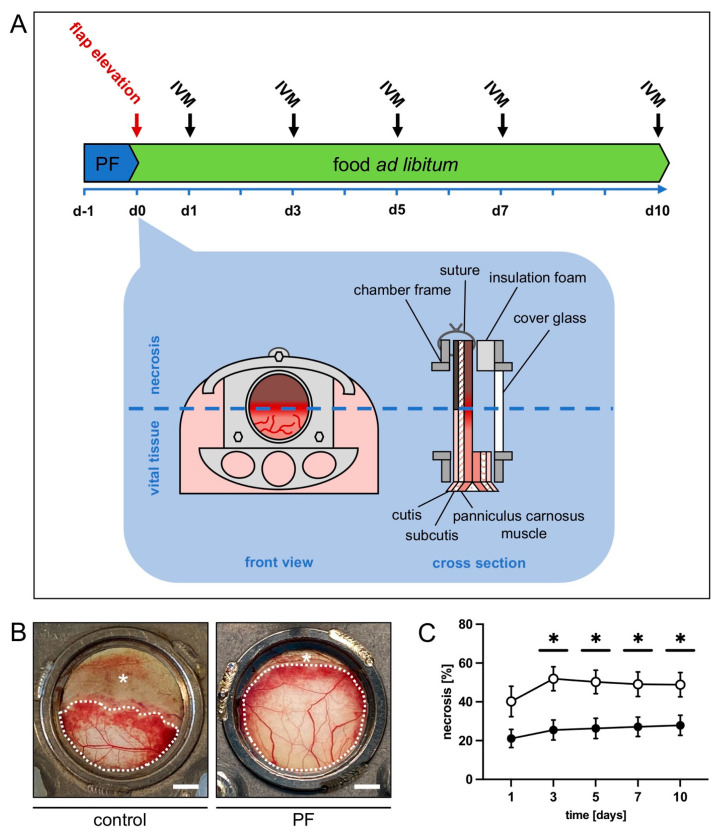
(**A**) Experimental protocol. Periodic fasting (PF) in the form of a 24 h fast was applied before flap elevation to the treated animals (*n* = 8). Treated animals had free access to standard diet for the remainder of the experiment. Animals with unrestricted access to standard diet served as controls (*n* = 8). After flap elevation and dorsal skinfold chamber placement on day 0, repeated intravital fluorescence microscopy (IVM) on day 1, 3, 5, 7 and 10 was used to assess flap survival and vascularization. The dorsal skinfold chamber with the musculocutaneous flap (15 × 11 mm) fixed between the symmetric titanium frames allowed microscopic access to the flap tissue through the observation window. The flap tissue consisted of the panniculus carnosus muscle, subcutis and cutis. (**B**) Macroscopic images of the observation window of an untreated control mouse and a mouse undergoing periodic fasting (PF), displaying a marked difference in tissue necrosis (asterisks) on day 5 after flap elevation. Vital tissue is marked by dotted line. Scale bar: 2 mm. (**C**) Necrosis rate [%] of flaps in PF-treated mice (black circles, *n* = 8) and untreated controls (white circles, *n* = 8) on days 1, 3, 5, 7 and 10 after flap elevation, as assessed by intravital fluorescence microscopy and computer-assisted image analysis. Means ± SEM. * *p* < 0.05 vs. control.

Mice in the PF group (*n* = 8) were subjected to a 24 h fast prior to flap elevation (Figure 1A). To ensure that the PF animals were not consuming previously buried chow or mouse droppings they were placed in a new cage for the duration of the fast. Before and after the fast, the animals had free access to standard pellet chow. Mice in the control group (*n* = 8) had free access to standard pellet chow during the entire observation period.

### 2.3. Anesthesia

For surgical flap elevation with dorsal skinfold chamber implantation and subsequent intravital fluorescence microscopy animals were put under general anesthesia by intraperitoneal injection of ketamine (100 mg/kg body weight; Ursotamin^®^; Serumwerke Bernburg, Bernburg, Germany) and xylazine (12 mg/kg body weight; Rompun^®^; Bayer, Leverkusen, Germany). All animals also received postoperative pain medication by means of subcutaneous injections of buprenorphine hydrochloride (0.01 mg/kg body weight; Temgesic^®^; RB Pharmaceuticals Limited, Slough, UK).

### 2.4. Dorsal Skinfold Chamber Flap Model

As described in detail in previous studies, a musculocutaneous flap with a random pattern design was raised on the back of each animal [24]. The flap was mounted into a dorsal skinfold chamber (Irola Industriekomponenten GmbH & Co. KG, Schonach, Germany) to enable repeated intravital fluorescence microscopy for microcirculation analysis within the flap tissue (Figure 1A). Depilation of the dorsum was performed. Then, a dorsal skin flap measuring 15 mm (base) × 11 mm (length) was elevated perpendicular to the animal’s spine. The flap was fixed back to the lateral wound margins with interrupted sutures and mounted between the two chamber frames. Insulation foam was used to ensure air tightness of the chamber. The flap tissue within the observation window was sealed using a cover glass and snap ring to make it accessible for repeated microscopic imaging. Due to the chosen width-to-length ratio the distal area of the flap was subjected to acute persistent ischemia as soon as flap elevation was performed. Over time, the tissue develops roughly 50% necrosis if kept untreated (Figure 1A,B). After the surgical preparation, the animals were allowed to recover for 24 h before the first microscopy. All animals tolerated the surgical flap elevation and the chamber implantation well, as shown by normal feeding and sleeping habits during the in vivo observation period.

### 2.5. Intravital Fluorescence Microscopy

On days 1, 3, 5, 7 and 10 after flap elevation, intravital fluorescence microscopy was carried out (Supplementary Material Figure S1). For this purpose, the animals were put under general anesthesia and fixed on a plexiglass platform to secure the observation window in a horizontal position. The mice received 0.1 mL 5% fluorescein isothiocyanate (FITC)-labeled dextran (150,000 Da; Sigma-Aldrich, Taufkirchen, Germany) plasma marker for contrast enhancement by means of injection into the retrobulbar veinous plexus. The chamber window was then placed under a Zeiss Axiotech fluorescence epi-illumination microscope (Zeiss, Oberkochen, Germany) to record flap microcirculation with a video camera (FK6990; Pieper, Schwerte, Germany) and a DVD system. Room temperature was kept at ~22 °C to avoid hypothermia of the animals during anesthesia. At the beginning of every microscopy, a panoramic image of the observation chamber was recorded for subsequent planimetric quantification of the perfused tissue surface. Regions of interest (ROI) were chosen in three distinct observational zones, proximal, medial and distal to the flap base. Thus, the chamber window was subdivided into three equal parts before selecting two ROI per zone. Each ROI was chosen containing an arterio-venous bundle or crossing that could be identified during each microscopy by its characteristic morphology for repeated measurements. Two capillary fields adjacent to the arterio-venous bundle were recorded per ROI. Necrotic ROI were documented with microscopic images as long as the non-perfused arterio-venous bundles could safely be identified. Additionally, one ROI within the medial transition zone between perfused and non-perfused tissue was recorded to track the formation of new blood vessels.

All parameters were analyzed using the offline analysis system CapImage (Version 8.5, Zeintl, Heidelberg, Germany). The rate of necrotic flap surface was determined as 100 − (perfused surface area/total chamber surface area × 100) and expressed in %. The functional capillary density (FCD) expressed in cm/cm^2^ was quantified per capillary field. In arterioles, capillaries and venules within each ROI microhemodynamic parameters were examined. Diameters of the vessels (*D*) were measured perpendicular to the vessel path in µm. The centerline red blood cell (RBC) velocity (*V*) was measured using the line shift method [25]. The volumetric blood flow (*VQ*) expressed in pL/s was calculated from *V* and *D* as $VQ=\pi \times {\left(\frac{D}{2}\right)}^{2}\times \frac{V}{K}\;$ with *K* (=1.6) representing the Baker–Wayland factor considering the parabolic velocity profile of blood in microvessels [26]. The density of newly formed microvessels within the transition zone was quantified and expressed in cm/cm^2^ to assess angiogenesis. Newly formed microvessels were distinguished by their irregular and entangled conformation as opposed to the straight, parallelly arranged capillaries of the panniculus carnosus muscle [27].

### 2.6. Histology and Immunohistochemistry

Tissue samples of the harvested flap tissue were fixed in formalin, embedded in paraffin and then cut into 3 µm-thick sections. Hematoxylin and eosin (HE) staining of all sections was carried out according to a standard protocol for initial assessment. All sections were analyzed using a BX60 microscope (Olympus, Hamburg, Germany) and the imaging software cellSens Dimension (Version 1.11, Olympus, Hamburg, Germany).

Individual sections were used for the immunohistochemical detection of myeloperoxidase-positive (MPO^+^) neutrophilic granulocytes and cleaved caspase (Casp)-3^+^ cells undergoing apoptosis. Citrate buffer was used to demask antigens in all samples. The unspecific binding sites were subsequently blocked using goat serum. Cell staining was performed by means of incubation with a polyclonal rabbit antibody against MPO (1:100; Abcam, Cambridge, UK) or a monoclonal rabbit antibody against Casp-3 (1:100; Cell signaling Technology, Danvers, MA, USA) as primary antibodies. A biotinylated goat anti-rabbit IgG antibody (ready-to-use; Abcam) was used as a secondary antibody. For the detection of the biotinylated antibody, peroxidase-labeled streptavidin (ready-to-use; Abcam) was applied and 3-amino-9-ethylcarbazole (Abcam) was used as a chromogen. Counterstaining was performed using Mayer’s hemalum (Merck, Darmstadt, Germany). The detected cells were quantified in two randomized high-power fields (HPFs) in the proximal and medial transition zones of the flaps. Distal necrotic tissue was excluded from analysis.

### 2.7. Statistical Analysis

All data was tested for normal distribution and equal variance. Differences between the two groups were analyzed by the unpaired Student’s *t*-test (GraphPad Prism 9; GraphPad Software, San Diego, CA, USA) or the Mann–Whitney rank sum test in case of non-parametric data. Statistical significance was accepted for a value of *p* < 0.05. In the following, all values are expressed as means ± standard error of the mean (SEM).

## 3. Results

### 3.1. Intravital Fluorescence Microscopy

The analysis of flap survival and vascularization in the murine dorsal skinfold chambers was conducted by means of repeated intravital fluorescence microscopy. Over the course of the 10 day observation period, PF-treated mice showed a significantly lower flap necrosis rate of 21–28% when compared to untreated control mice that exhibited a necrosis rate of 40–51% (Figure 1B,C). Notably, both groups experienced a slight increase in the necrosis rate from day 1 to 3, as blood flow ceased completely in critically perfused distal flap zones. This increase was more noticeable in the control group (Figure 1C).

**Figure 2:**
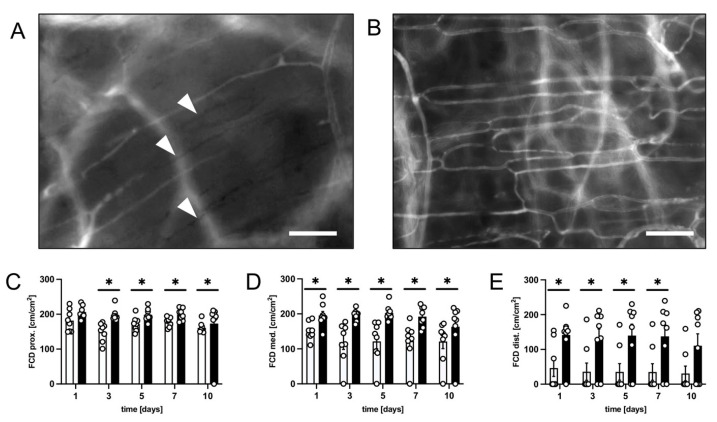
(**A**) Intravital fluorescent microscopic image of a critically perfused capillary bed in the distal flap tissue of a control animal 5 days after flap elevation. Capillaries that were no longer perfused are marked with white arrowheads. Scale bar: 50 µm. (**B**) Intravital fluorescent microscopic image of a well-perfused capillary bed in the distal flap tissue of a PF-treated animal on day 5 after flap elevation. Scale bar: 50 µm. (**C**–**E**) FCD [cm/cm^2^] in the proximal (**C**), medial (**D**) and distal zone (**E**) of flaps in PF-treated mice (black bars, *n* = 8) and untreated controls (white bars, *n* = 8) on days 1, 3, 5, 7 and 10 after flap elevation, as assessed by intravital fluorescence microscopy and computer-assisted image analysis. Means ± SEM. * *p* < 0.05 vs. control.

The improved flap survival rate in PF-treated animals was associated with a significantly higher FCD in all flap zones throughout the observation period (Figure 2A–E). The proximal and medial zones of the flaps in PF-treated animals exhibited a FCD of ~160–200 cm/cm^2^ (Figure 2C,D), while the distal zone had a reduced FCD of ~110–140 cm/cm^2^ (Figure 2E). In contrast, the FCD in the proximal and medial zones of the flaps in untreated mice was significantly lower with ~120–180 cm/cm^2^, and even as low as ~35 cm/cm^2^ in the distal zone (Figure 2C–E).

In addition, the diameters and centerline RBC velocities in arterioles, capillaries and venules of the flaps were recorded and VQ was calculated. In both groups, values increased in all vessel types over the observation period in response to the changed arterial inflow (Table 1). Moreover, VQ of vessels in PF-treated animals was higher when compared to controls.

**Figure 3:**
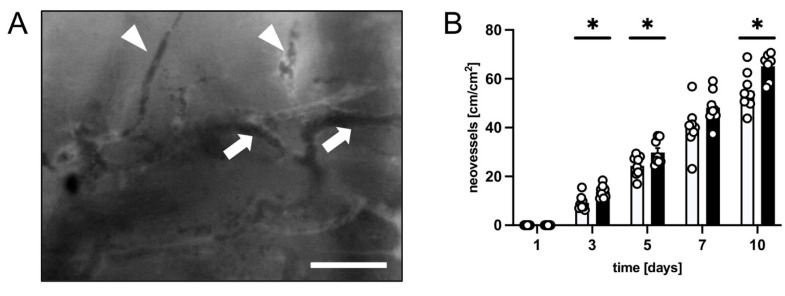
(**A**) Intravital fluorescent microscopic image showing vessel sprouts (marked by white arrowheads) originating from dilated capillaries (marked by white arrows) in the transition zone of a flap in a PF-treated mouse on day 10 after flap elevation. Scale bar: 50 µm. (**B**) Neovessels [cm/cm^2^] in the medial transition zone of flaps in PF-treated mice (black bars, *n* = 8) and untreated controls (white bars, *n* = 8) 1, 3, 5, 7 and 10 days after flap elevation, as assessed by intravital fluorescence microscopy and computer-assisted image analysis. Means ± SEM. * *p* < 0.05 vs. control.

Throughout the 10 day observation period, the development of new blood vessels in the transition zone between viable flap tissue and necrosis was quantified (Figure 3). In both groups, the flap tissue in this zone showed characteristic changes in capillary architecture starting from day 3 to 5. Capillaries exhibited irregular diameters due to vessel dilation and the formation of angiogenic vessel buds and sprouts, which grew out of preexisting microvessels (Figure 3A). The density of new microvessels was markedly higher in the flaps of PF-treated mice between days 5 and 10 when compared to the flaps in control animals (Figure 3B).

### 3.2. Histological and Immunohistochemical Analysis

**Figure 4:**
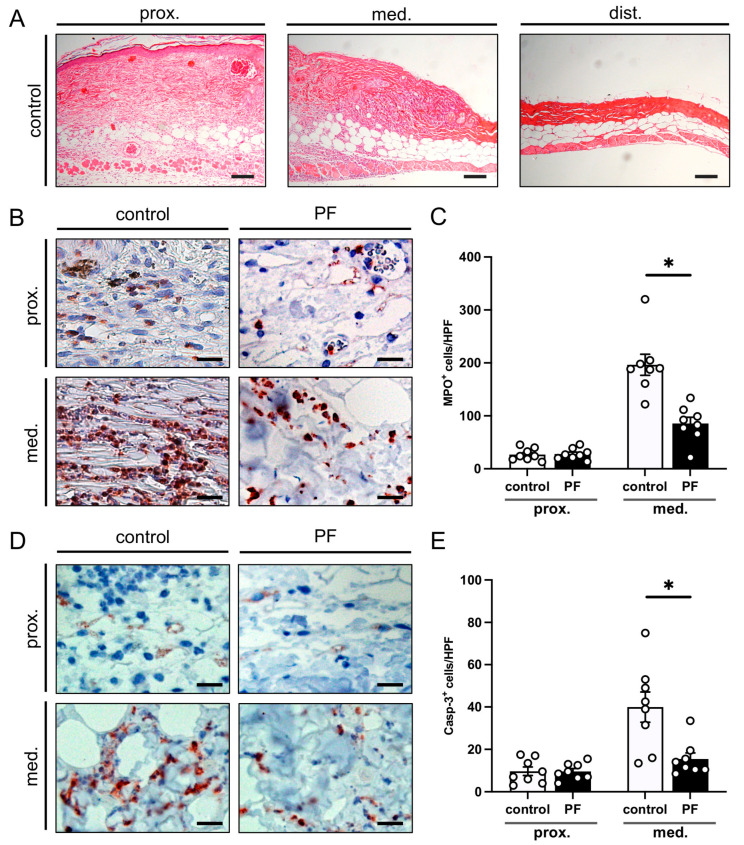
(**A**) Microscopic images of HE-stained sections of the proximal, medial and distal zone of an untreated flap in a control animal on day 10 after flap elevation. The proximal flap base consisted of vital cutis, subcutis and the underlying panniculus carnosus muscle. In the medial zone, these layers were disrupted and the tissue was infiltrated by inflammatory cells. The distal tissue zone was completely necrotic and showed a lack of cell nuclei and a reduced thickness. Scale bar: 100 μm. (**B**) Microscopic images of immunohistochemically stained sections of the proximal and medial zone of flaps in an untreated control mouse and a mouse undergoing periodic fasting (PF) 10 days after flap elevation. The sections were stained with an antibody against the neutrophilic granulocyte marker MPO. Scale bars: 20 µm. (**C**) MPO^+^ cells/HPF in the proximal and medial zones of flaps in PF-treated mice (PF; black bars, *n* = 8) and untreated controls (control; white bars, *n* = 8) on day 10 after flap elevation, as assessed by immunohistochemistry. (**D**) Microscopic images of immunohistochemically stained sections of the proximal and medial zone of flaps in an untreated control animal and an animal undergoing periodic fasting (PF) 10 days after flap elevation. The sections were stained with an antibody against the apoptosis marker Casp-3. Scale bars: 20 µm. (**E**) Casp-3^+^ cells/HPF in the proximal and medial zones of flaps in PF-treated mice (PF; black bars, *n* = 8) and untreated controls (control; white bars, *n* = 8) on day 10 after flap elevation, as assessed by immunohistochemistry. Means ± SEM. * *p* < 0.05 vs. control.

After the in vivo observation period, histological analyses were performed to examine morphological changes within the flap tissue. The transition zone between the proximal vital flap tissue and necrotic distal areas was identified using HE-stained sections (Figure 4A). The distal zone was completely necrotic and was therefore excluded from further immunohistochemical analyses.

The analysis of MPO^+^ neutrophilic granulocytes revealed a significant invasion of these immune cells in the medial transition zones of the flaps in both PF-treated and untreated mice, which was not the case in the proximal zone (Figure 4B,C). The inflammatory tissue reaction was significantly decreased in PF-treated animals, as indicated by a significantly reduced number of MPO^+^ cells/HPF when compared to control mice (Figure 4C). In addition, apoptotic cells were identified using immunohistochemical Casp-3 staining. In both groups, the proximal vital zone of the flaps exhibited only a few apoptotic cells, whereas more Casp-3^+^ cells could be detected in the medial transition zone (Figure 4D,E). However, PF treatment led to a significant reduction in the number of Casp-3^+^ cells/HPF in the flap tissue of the transition zone when compared to controls (Figure 4D,E).

## 4. Discussion

Potential benefits of long-term DR, such as life span extension, are considered irrelevant in a clinical setting, because most patients struggle to adhere to a restricted diet for longer periods of time [28]. Moreover, longer fasting protocols may drastically complicate the scheduling of elective surgeries. However, even short-term DR increases acute stress resistance [15,29] and, thus, has been proposed as an approach to counteract surgical stress and ischemia [9]. In fact, previous studies show that short periods of DR are sufficient to trigger tissue-protective cellular mechanisms [9,15]. These mechanisms were also observed in aged and overweight mice and have been suggested to be age-independent [30,31]. This indicates that even challenging patient groups with comorbidities or older patients may benefit from preoperative DR. Currently, the only dietary recommendation prior to surgery is fasting overnight in order to avoid aspiration of regurgitated food under anesthesia. However, a short extension of the fast may already be enough to utilize the tissue-protective mechanisms attributed to PF for the reduction in ischemia-induced complications in flap surgery.

With the present research, we were able to prove that the rate of flap survival is significantly increased by a short-term preoperative fast of 24 h. Importantly, this time period is short enough to be easily implemented in a clinical setting and has already been shown to induce beneficial effects in previous studies regarding DR [21,32]. The used 24 h PF regimen suppressed ischemia-induced inflammation and maintained nutritive tissue perfusion.

To investigate the effect of PF on the overall survival of flaps and their changes in microcirculation, a dorsal skinfold chamber model was used in combination with a musculocutaneous random pattern flap with clearly defined dimensions [24]. This approach bears the major advantage that microvascular flap perfusion and blood vessel formation can be repeatedly analyzed over the course of 10 days by means of intravital fluorescence microscopy, both morphologically and dynamically. In the present study, tissue necrosis was only evaluated by means of intravital fluorescence microscopy. Further studies should also include the analysis of relevant biomarkers, to clarify the involved cellular mechanisms. Further limitations of this study include the relatively small number of animals and the fairly short 10 day follow up period. Moreover, there are limited data regarding the question how a certain length of fasting in mice compares to the same period in humans. Changes in protein acetylation associated with autophagy as an effect of fasting could be detected in circulating white blood cells in both mice and humans [33]. However, four days of fasting was necessary in humans, while 48 h of fasting was sufficient in mice to measure the onset of these effects [33]. Thus, further research would need to clarify the exact length of the preoperative fasting period needed to improve flap survival in humans.

Several mechanisms seem to add up to the beneficial effects of DR on ischemic tissue in a synergistic manner. DR does not only induce stress resistance to nutrient deprivation, but also causes a cross-resistance against other stressors, such as oxidative insults [34]. This effect is mediated by an increased expression of cytoprotective genes, such as hemeoxygenase-1 and components of the glutathione detoxification system [21]. Furthermore, it has been suggested that cells shift into survival and maintenance programs under fasting conditions to slow down their metabolism, which may help protect them from the ischemic insult [35]. By drastically reducing the number of cell divisions during the fast, cells can utilize metabolites originating from the breakdown of fats, proteins and organelles and become more stress resistant. This effect has been investigated extensively in the context of chemotherapy. As cancer cells are not able to enter a non-dividing and protected state due to the acquired self-sufficiency in growth signaling, they remain sensitive to chemotherapeutic agents, while the cellular resistance of healthy cells can be drastically increased by means of DR [9,35,36]. This increased cellular resistance could also be seen in the present study, as indicated by a significantly lower number of apoptotic cells in the transition zone of flaps in PF-treated animals and an overall increase in flap tissue survival.

Another mechanism that may be particularly beneficial in the context of flap surgery is the reduction in oxidative stress and inflammation through DR [29]. Several biomarkers for oxidative stress in the heart, brain and kidneys of rabbits were reduced after short-term IF or PF [37]. Moreover, fasted rats displayed a reduced expression of pro-inflammatory cytokines and chemokines, such as interleukin-1β, tumor necrosis factor-α and monocyte chemoattractant protein-1, in multiple tissues, including liver, kidney and spleen [38]. In line with these findings, we detected significantly less invading neutrophilic granulocytes in the transition zone of flaps in PF-treated animals when compared to controls. Suppressing the invasion of neutrophils is crucial for flap survival, as the cells not only cause parenchymal cell dysfunction, but also negatively impact blood rheology [39,40].

It has to be considered that sufficient nutrition is of central importance for tissue regeneration [41]. However, it has been shown in rodent models that short-term preoperative DR followed by free food access after surgery has protective effects even in highly proliferative tissues, including the intestine [42]. Accordingly, we also did not observe a weaker angiogenic response in animals treated with fasting, although the process of blood vessel development crucially relies on the proliferation of endothelial cells. Interestingly, PF-treated animals even showed a significantly increased formation of microvessels in the transition zone between vital and necrotic tissue. This finding may be explained by the overall improved tissue viability in flaps of PF-treated mice, which may result in a higher capacity to react to the ischemic stimulus.

## 5. Conclusions

In conclusion, the present study shows that a 24 h fast immediately before flap elevation promotes flap tissue survival by maintaining nutritive tissue perfusion and suppressing ischemia-induced inflammation. In contrast to other conditioning strategies, PF may be easily implemented into standard clinical protocols, as it is quick and easy to perform without additional costs. Moreover, it is a safe approach, which does not induce severe side effects. Hence, PF is a highly promising approach for translation into clinical practice, which may markedly contribute to an increase in the future success rates of elective flap surgery.

## Figures and Tables

**Table 1:** Volumetric blood flow [pL/s] of arterioles, capillaries and venules in the three zones (proximal, medial and distal) of flaps in untreated control mice (*n* = 8) and mice undergoing periodic fasting (PF; *n* = 8) 1, 3, 5, 7 and 10 days after flap elevation, as assessed by intravital fluorescence microscopy and computer-assisted image analysis.

Volumetric Blood Flow [pL/s]		d1	d3	d5	d7	d10
Arterioles						
prox. control		348 ± 53	404 ± 44	554 ± 91	767 ± 130	1019 ± 150
	PF	643 ± 126 *	1165 ± 178 *	1438 ± 282 *	1637 ± 209 *	1812 ± 178 *
med. control		325 ± 98	389 ± 104	530 ± 124	770 ± 203	1087 ± 247
	PF	742 ± 149 *	1367 ± 230 *	1523 ± 289 *	1907 ± 301 *	2000 ± 269 *
dist. control		271 ± 45	341 ± 193	603 ± 4	478 ± 203	640 ± 219
	PF	70 ± 157	931 ± 344	1496 ± 342	1375 ± 399	1610 ± 469
Capillaries						
prox. control		1 ± 0	2 ± 0	3 ± 0	4 ± 1	6 ± 1
	PF	2 ± 0 *	4 ± 1 *	6 ± 1 *	9 ± 1 *	13 ± 2 *
med. control		1 ± 0	2 ± 0	3 ± 0	4 ± 1	5 ± 1
	PF	2 ± 0	4 ± 0 *	6 ± 1 *	10 ± 1 *	12 ± 2
dist. control		1 ± 0	2 ± 1	2 ± 0	4 ± 1	5 ± 0
	PF	1 ± 0	3 ± 1	5 ± 1 *	9 ± 3	11 ± 2 *
Venules						
prox. control		441 ± 92	679 ± 161	1304 ± 307	1872 ± 452	2154 ± 491
	PF	881 ± 287	1377 ± 261 *	1500 ± 256	2568 ± 508	3060 ± 567
med. control		424 ± 116	537 ± 115	1020 ± 166	1675 ± 280	2713 ± 409
	PF	539 ± 157	1443 ± 446 *	1692 ± 262	3125 ± 533 *	3767 ± 558
dist. control		197 ± 107	577 ± 131	637 ± 203	1184 ± 155	1565 ± 137
	PF	514 ± 153	1088 ± 310	1768 ± 460	2650 ± 884	2484 ± 514

Mean ± SEM. * *p* < 0.05 vs. control.

## Data Availability

All discussed data can be obtained from the manuscript.

## Supplementary Materials

The following supporting information can be downloaded at: https://www.mdpi.com/article/10.3390/biomedicines12030690/s1, Figure S1: Exemplary macroscopic image (A) of an untreated control flap with the corresponding intravital fluorescent microscopic image (B) on day 1 after flap elevation. Scale bars: 2 mm.
