# Supplementary material for: Short-Term Periodic Fasting Reduces Ischemia-Induced Necrosis in Musculocutaneous Flap Tissue
Source: Biomedicines. 2024 Mar 20;12(3):690. doi: 10.3390/biomedicines12030690 (PMC10968411; doi:10.3390/biomedicines12030690)
Supplement: Supplementary file 1 [file biomedicines-12-00690-s001.zip › biomedicines-2871259-supplementary.pdf]

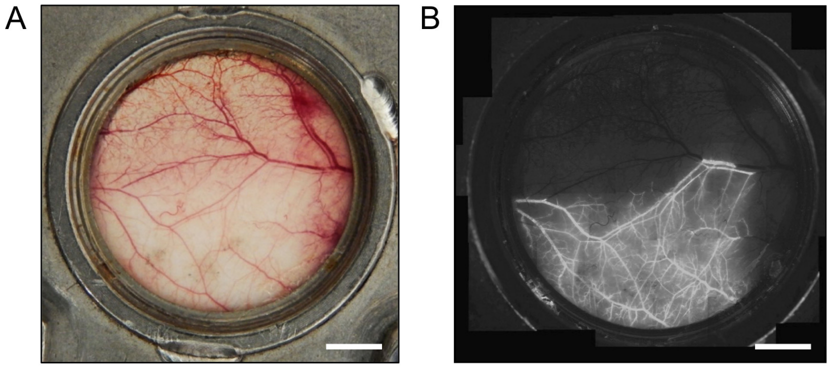

Supplementary Material Figure S1. Exemplary macroscopic image (A) of an untreated control flap with the corresponding intravital fluorescent microscopic image (B) on day 1 after flap elevation. Scale bars: 2 mm.
